# Chimeric Antigen Receptor T-cell (CAR T) Therapy for Hematologic and Solid Malignancies: Efficacy and Safety—A Systematic Review with Meta-Analysis

**DOI:** 10.3390/cancers11010047

**Published:** 2019-01-07

**Authors:** Wen-Liang Yu, Zi-Chun Hua

**Affiliations:** 1 The State Key Laboratory of Quality Research in Chinese Medicine, Macau Institute for Applied Research in Medicine and Health, Macau University of Science and Technology, Avenida Wai Long, Taipa, Macao 999078, China; q1050574228@163.com; 2 Jiangsu Target Pharma Laboratories Inc., Changzhou High-Tech Research Institute of Nanjing University, Changzhou 213164, China; 3 School of Life Sciences, Nanjing University, Nanjing 210023, China; 4 Shenzhen Research Institute of Nanjing University, Shenzhen 518057, China

**Keywords:** chimeric antigen receptor T cell, response, side effect, relapse, subgroup analysis, meta-analysis, meta-regression analysis

## Abstract

Chimeric antigen receptors T cells (CAR T) had been used for treating various tumor patients in clinic, and owned an incredible efficacy in part of malignancies. However, CAR T therapy remains controversial due to doubts about its efficacy and safety in the clinical treatment of various malignancies. A total of 997 tumor patients from 52 studies were included in this review. Eligible studies were searched and reviewed from the databases of PubMed, Web of Science, Wanfang and Clinicaltrials.gov. Then meta-analysis and subgroup analysis were used to investigate the overall response rate (ORR), complete response rate (CRR), common side effect rate (CSER) and relapse rate (RR) of CAR T therapy for patients in clinical researches, respectively. The results further confirmed that CAR T therapy had a higher response rate for hematologic malignancies. More importantly, CAR T therapy had a higher CSER in patients with hematologic malignancies, and it had a similar RR in patients with different malignancies. Cell cultured without the addition of IL-2 and total administration less than 10^8^ cells were recommended. This study offers a reference for future research regarding the application in solid and hematologic malignancies, side effects and relapse, and even the production processes of CAR T cells.

## 1. Introduction

In recent decades, immune-based therapies have made some extraordinary strides in clinical trials, offering the possibility of achieving long-term remission and even complete cure for tumor patients [1,2]. Chimeric antigen receptors (CARs)-based T cell adoptive immunotherapy, as a personalized targeted immunotherapy option, has become more and more popular in the war against tumors [3,4]. CARs are a type of antigen-targeted receptor, which combine extracellular tumor-binding moieties with intracellular T-cell signaling domains. The most commonly one is single-chain variable fragment (scFv), which comes from monoclonal antibodies. CARs are allowed to use a single receptor construct for any given antigen in all patients, because it directly recognizes cell surface antigens, and it is independent of MHC-mediated expression [5]. Since the CAR-engineered T cells targeting CD19 (CD19 CAR T) were successfully used in B-cell lymphoma in 2010 [6], various types of CAR T cells have been constructed by scientists, and applied to tumor treatment in clinical trials.

At present, first- second- third- fourth- generation CARs have been produced and tested in clinical trials. The first generation CARs consist of a scFv antibody, used for target binding, connected by a spacer domain, generally the IgG1 CH1CH2, to the transmembrane signaling domain of CD3ζ obtained from the T cell receptor. They have limitations in amplification, persistence, and antitumor efficacy in vivo [6,7,8]. The second generation CARs added costimulatory molecules (such as CD28, 4-1BB or OX40) to provide second signals [8]. The third generation CARs were series connected with multiple costimulatory domains [9]. It has been confirmed that the second and third generation CARs could improve efficacy in some tumors [10]. The main side effects caused by CAR T cells include cytokine release syndrome (CRS), graft-versus-host disease (GVHD), neurologic symptoms (NS) and tumor lysis syndrome (TLS) [11], among them, GVHD only happens when using an allogeneic CAR T without previous T cell receptor deletion. The fourth generation CARs (termed TRUCK T cells) are different from first- second- third- generation CARs. They were engineered to express cytokines (particularly IL-12) that could be used to regulate the antitumor immunologic microenvironment [12]. Besides, it is generally believed that CAR T cells with higher doses and longer persistence in vivo can lead to better response rates and lower RR, respectively.

CD19 CAR T cells have shown a stable antitumor efficacy in patients with recurrent B cell acute lymphoblastic leukemia (B-ALL), CLL, and non-Hodgkin’s lymphoma (NHL) in recent years [13]. As an immunotherapy method, CAR T therapy has not only had remarkable efficacy in several hematologic malignancies, but has also achieved a certain efficacy for many other different hematologic and solid malignancies in clinical trials, such as glioblastoma (GBM), neuroblastoma (NB), non-small cell lung cancer (NSCLC), osteosarcomas (OS), Ewing sarcoma (EWS), etc. With the increasing number of studies, different questions about the clinical efficacy and safety of CAR T cells continue to emerge. Due to the high therapeutic threshold and the high cost of CAR T therapy, the clinical sample size is still limited so far, especially in the patients with solid malignancies. However, solid malignancies are regarded as the main research direction in current research. Therefore, a systematic analysis of CAR T therapy is necessary, especially of the response rate for patients with solid malignancies, CSER and RR. This study aims to provide a systematic review of CAR T therapy through meta-analysis, subgroup analysis and meta-regression analysis, offering a reference for its future application and research.

## 2. Results

### 2.1. Research Results and Quality Assessment

**Figure 1:**
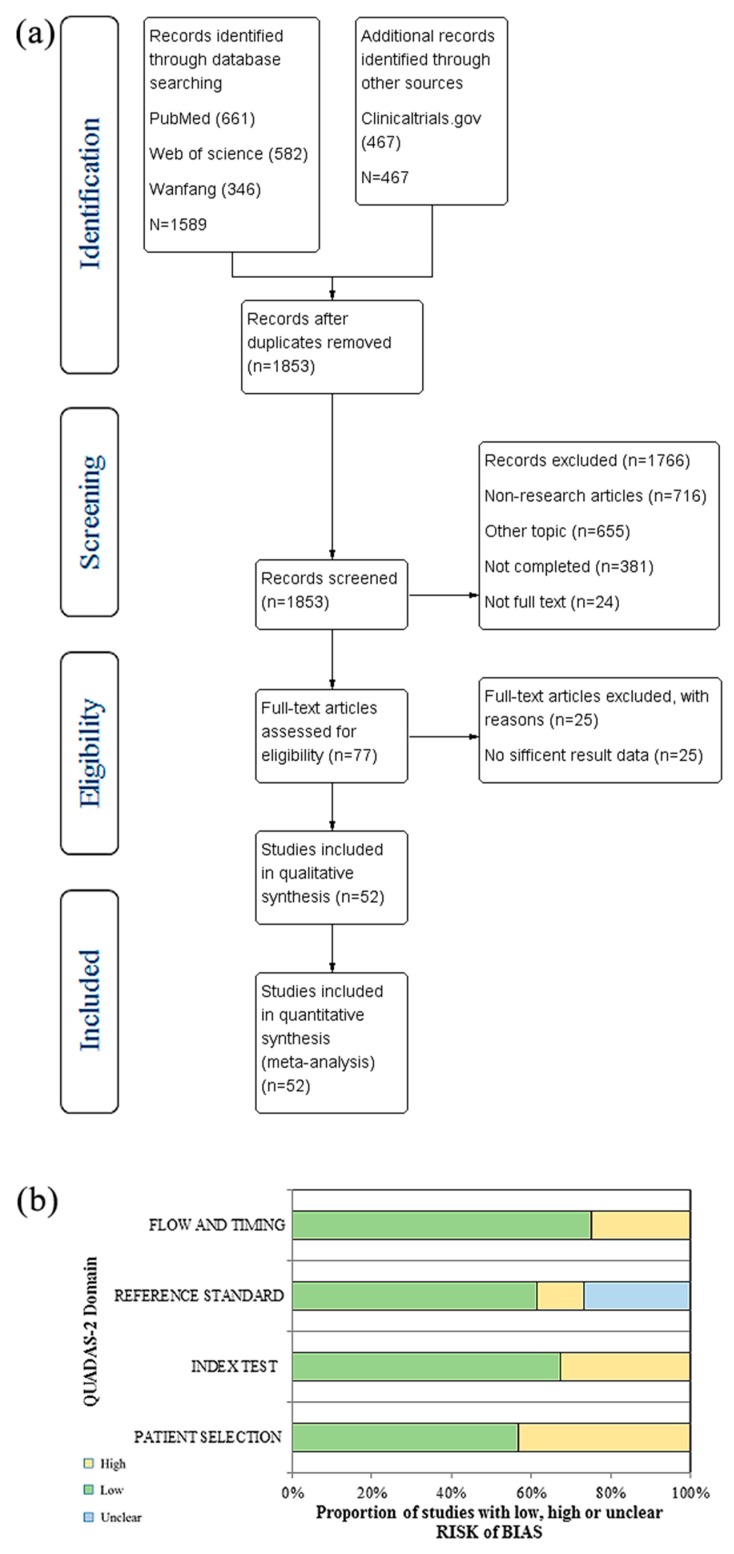
Research results and quality assessment: (**a**) Flow-diagram of the literature selection process; (**b**) Results of the methodologic assessment by using the Quality Assessment of Diagnostic Accuracy Studies (QUADAS-2) tools.

The literature search process was included in a PRISMA flow diagram (Figure 1a). By reviewing titles and abstracts the preliminary screening identified 1589 potentially relevant publications and 467 clinical reports. After removing duplicates, 1853 potential relevant publications and clinical reports were preliminarily included, including 716 non-research articles, 655 other topic articles, 381 uncompleted reports, and 24 not full-text articles. Then we further screened the remaining 77 full-text articles, and 25 articles with insufficient result data were excluded. Finally, 52 studies [14,15,16,17,18,19,20,21,22,23,24,25,26,27,28,29,30,31,32,33,34,35,36,37,38,39,40,41,42,43,44,45,46,47,48,49,50,51,52,53,54,55,56,57,58,59,60,61,62,63,64,65] was included in our research, including fifty-one English studies [14,15,16,17,18,19,20,21,22,23,24,25,26,27,28,29,30,31,32,33,34,35,36,37,38,39,40,42,43,44,45,46,47,48,49,50,51,52,53,54,55,56,57,58,59,60,61,62,63,64,65] and one Chinese study [41].

According to the QUADAS-2 user guidelines, the result of quality evaluation for 52 included studies was shown in Table 1. And the proportion of studies with low, high or unclear risk of bias were shown in Figure 1b. The agreement for selection of studies between two authors was high.

### 2.2. Characteristics of Studies

Fifty-two studies, with a total of 997 patients were included in our studies. The basic characteristics of patients are shown in Table 1. Twenty (20) kinds of CAR T cells were included, including CAR-modified T cell-epidermal growth factor receptor variant III (EGFRvIII), CAR T cells specific for GD2 (GD2-specific CAR T), IL-13 receptor α2-Redirected CAR CD8+ T (IL13Rα2-specific CD8+ CAR T), CAR T-epidermal growth factor receptor (CAR T-EGFR), human epidermal growth factor receptor 2-CAR T(HER2-CAR T), carbonic anhydrase IX-CAR T (CAIX-CAR T), CART-mesothelin (CART-meso), anti-Mucin 1 CAR T (anti-MUC1 CAR T), carcinoembryonic antigen CAR T (CEA CAR T), anti-prostate specific membrane antigen designer CAR T (PSMA designer CAR T), mRNA CAR T cells directed against c-Met (mRNA c-Met-CAR T), CAR T cells targeting tumor-associated glycoprotein-72 (CART72), CAR-B-cell maturation antigenT (CAR-BCMAT), 19-28z-Toll-like receptor 2 CAR T (1928zT2 CAR T), CD19 CAR T, CD30-targeting CAR T (CART-30), CD22-targeted CAR T (CD22-CAR T), CD20-CAR-modified T (anti-CD20 CART), CD19-targeted 19-28z CAR T (19-28z CAR T), CAR T Cell Against the LeY Antigen (LeY CAR T). Most of patients were treated by autologous CAR T cells, only three patients were treated by donor-derived CAR T cells. Other information about the CAR T cells is shown in Table 2, including vectors, gene transduction method, T cell activate method, T cell culture time, total administration doses and persistence time in vivo. A total of 31 types of malignancies were diagnosed, including 21 types of solid malignancies [14,15,16,17,18,19,20,21,22,23,24,25,26,27,28,29,30,31,32,33] and 10 types of hematologic malignancies [34,35,36,37,38,39,40,41,42,43,44,45,46,47,48,49,50,51,52,53,54,55,56,57,58,59,60,61,62,63,64,65]. Among them, solid malignancies included colorectal cancer (CRC), desmoplastic small round cell tumor (DSRCT), Ewing sarcoma (EWS), GBM, malignant pleural mesothelioma (MPM), etc. 

Hematologic malignancies were mainly concentrated on lymphoma and leukemia, including acute lymphoblastic leukemia (ALL), splenic marginal zone lymphoma (SMZL), primary mediastinal large B-cell lymphoma (PMBCL), acute myeloid leukemia (AML), Hodgkin lymphoma (HL), mantle cell lymphoma (MCL), diffuse large B cell lymphomas (DLBCL) etc. Besides, 878 patients received chemotherapy-based lymphodepletion before CAR T therapy, 98 patients did not receive lymphodepletion, and 21 patients did not any information about lymphodepletion. Since no information on clinical response was provided for 156 patients, only 841 patients were eligible for the evaluation of response rate. And seven included studies did not offer any information about common side effects, thus 896 patients were eligible for the analysis of common side effects. Finally, 533 patients who achieved overall response after treatment were all included in the analysis of relapse.

### 2.3. Meta-Analysis of ORR of CAR T Therapy in Patients with Different Malignancies

**Figure 2:**
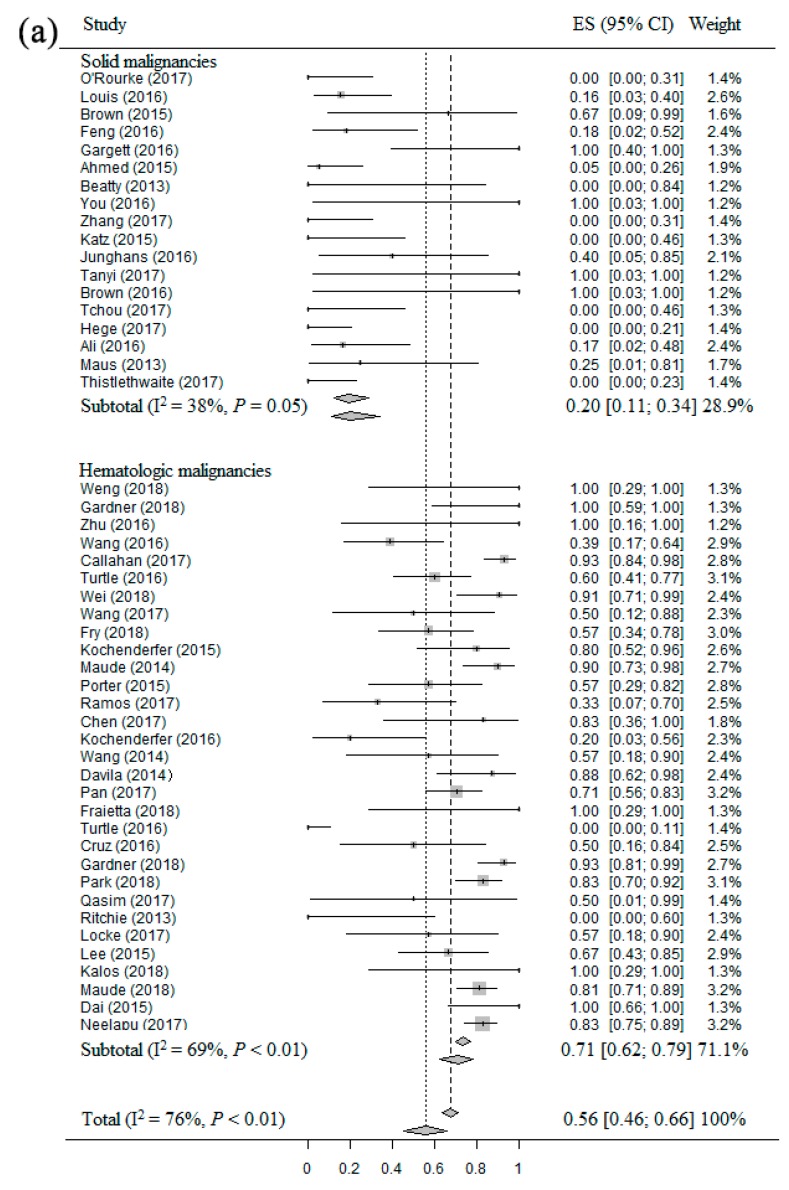

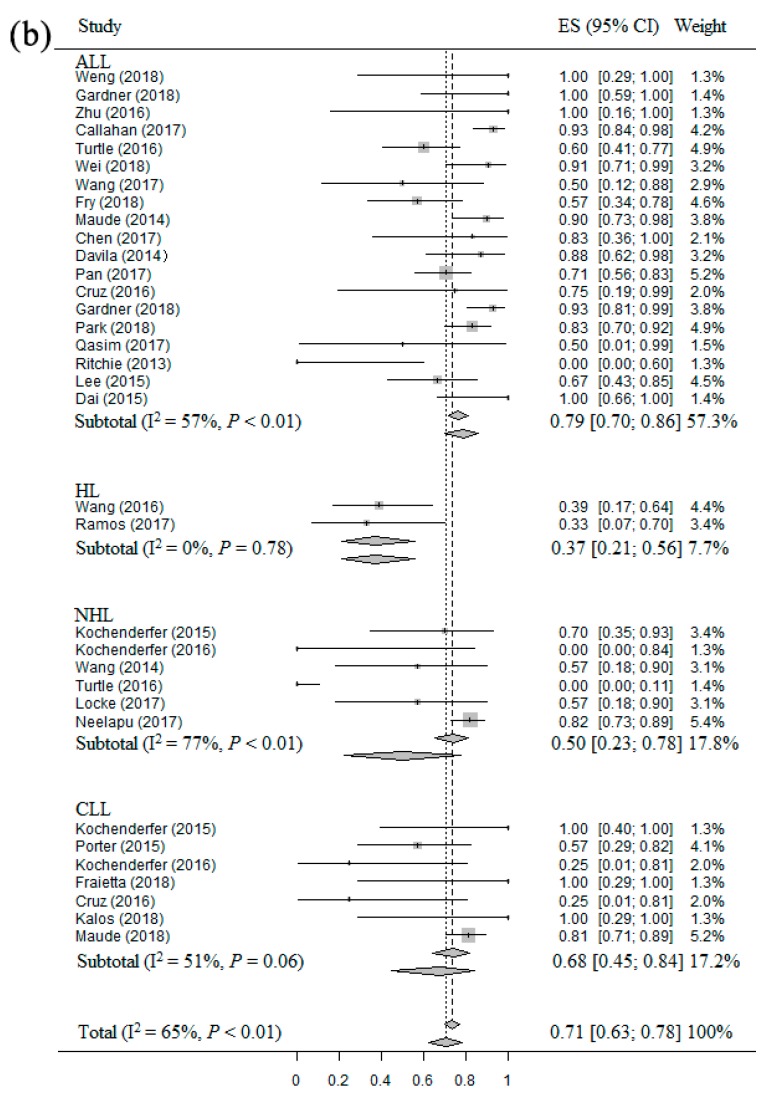
The forest plots of meta-analysis about ORR: (**a**) Forest plot for ORR and CI in solid and hematologic malignancies patients of each study and the overall; (**b**) Forest plot for ORR and CI in different B-cell malignancies patients of each study and the overall.

Forty-nine studies [14,15,16,17,18,19,21,22,23,24,25,26,27,28,29,30,31,32,34,35,36,37,38,39,40,41,42,43,44,45,46,47,48,49,50,51,52,53,54,55,57,58,59,60,61,62,63,64,65] including 841 patients were eligible for the ORR evaluation in patients with different malignancies. The ORR of CAR T therapy for patients with solid and hematologic malignancies in each clinical studies showed wide differences, from 0.0% to 100.0%. The overall estimate of ORR and 95% confidence interval (CI) from the individual studies is shown in Figure 2a. Homogeneity tests suggested that these studies had significant heterogeneity (I^2^ = 76%, *p* < 0.01). Thus, the random-effect model was used to calculate them. The overall pooled ORR of CAR T therapy for patients with solid and hematologic malignancies was 56% (95% CI: 46–66%). Based on the result of subgroup analysis, the ORR was significantly higher for patients with hematologic malignancies (71%, 95% CI: 62–79%) compared to patients with solid malignancies (20%, 95% CI: 11–34%). The result of subgroup analysis of the patients with B-cell malignancies (Figure 2b) showed that the ORR of patients with ALL, HL, NHL and CLL were 79% (95% CI: 70–86%), 37% (95% CI: 21–56%), 50% (95% CI: 23–78%) and 68% (95% CI: 45–84%), respectively.

### 2.4. Meta-Analysis of CRR of CAR T Therapy in Patients with Different Malignancies

**Figure 3:**
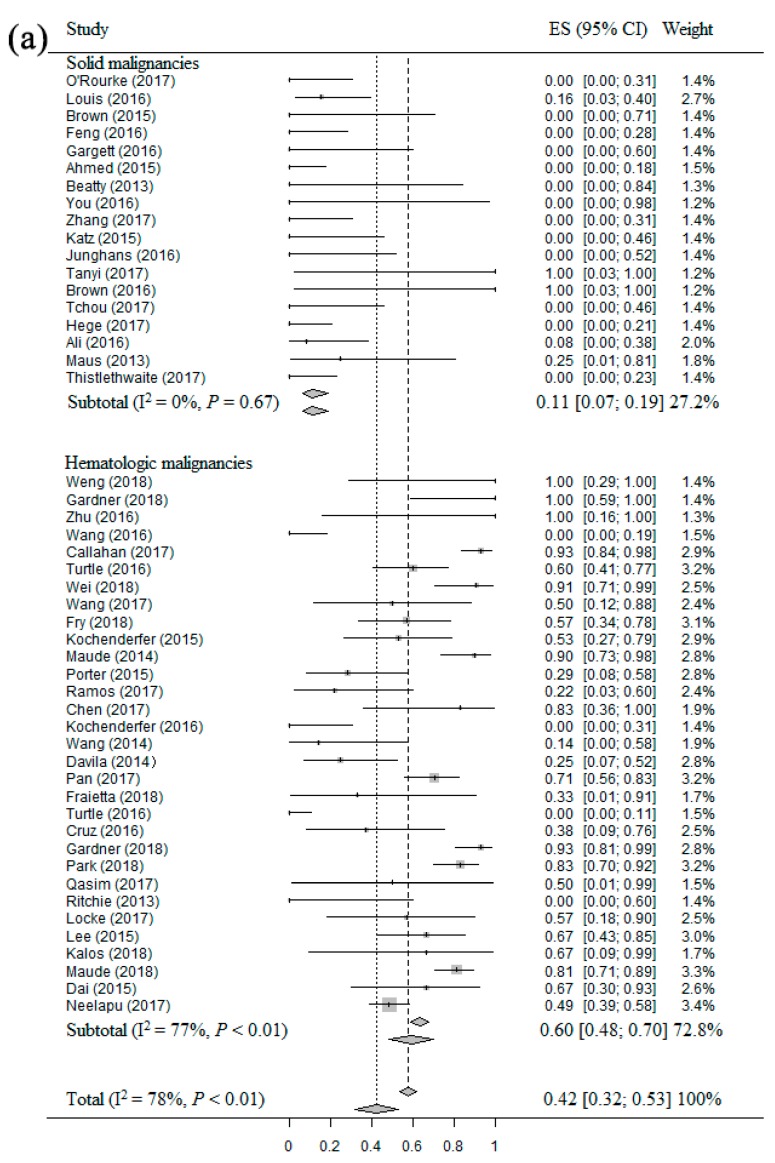

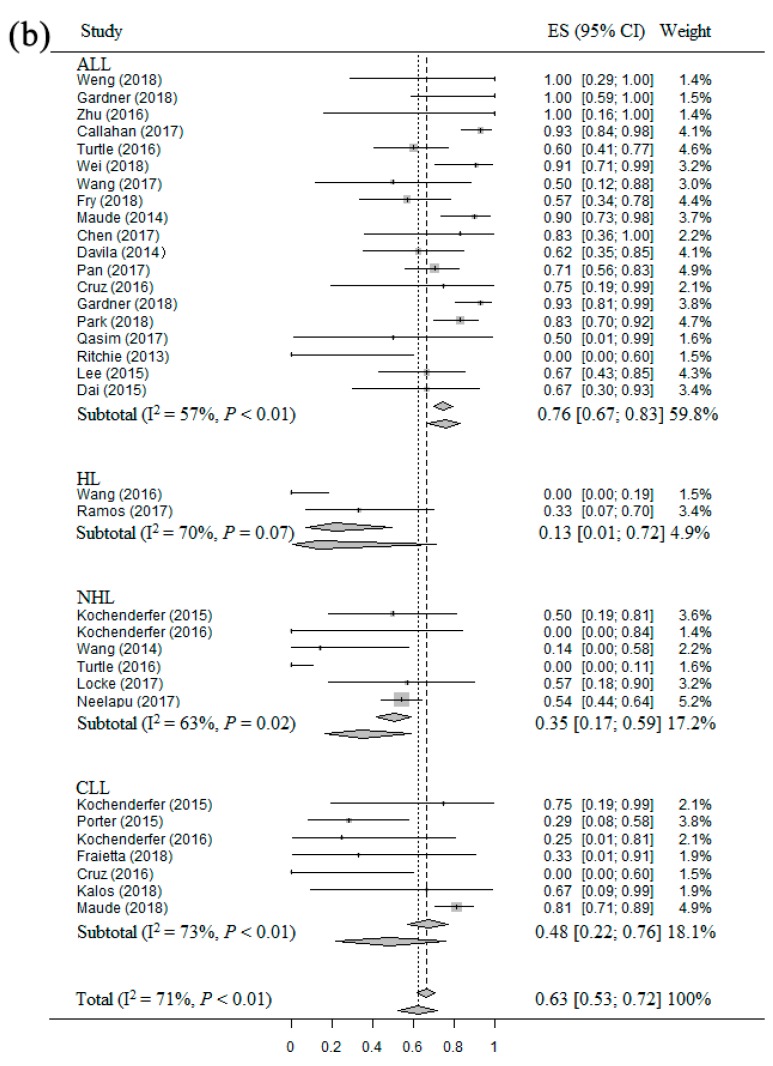
The forest plots of meta-analysis about CRR: (**a**) Forest plot for CRR and CI in solid and hematologic malignancies patients of each study and the overall; (**b**) Forest plot for CRR and CI in different B-cell malignancies patients of each study and the overall.

Forty-nine studies [14,15,16,17,18,19,21,22,23,24,25,26,27,28,29,30,31,32,34,35,36,37,38,39,40,41,42,43,44,45,46,47,48,49,50,51,52,53,54,55,57,58,59,60,61,62,63,64,65] including 841 patients were eligible for the CRR evaluation of CAR T therapy in patients with different malignancies. The CRR in each clinical study had wide differences as well (from 0.0% to 100.0%). The overall estimate of CRR and 95% CI from the individual studies were shown in Figure 3a. Homogeneity test appeared that these studies had significant heterogeneity (I^2^ = 78%, *p* < 0.01). Therefore, we used the random-effect model to calculate them. The overall pooled CRR of CAR T therapy for patients with solid and hematologic malignancies was 42% (95% CI: 32–53%). The subgroup analysis result showed that the CRR was significantly higher for patients with hematologic malignancies (60%, 95% CI: 48–70%) compared to patients with solid malignancies (11%, 95% CI: 7–19%). The subgroup analysis result regarding the patients with different B-cell malignancies (Figure 3b) showed that the CRR of patients with ALL, HL, NHL and CLL were 76% (95% CI: 67–83%), 13% (95% CI: 1–72%), 35% (95% CI: 17–59%) and 48% (95% CI: 22–76%), respectively.

### 2.5. Meta-Analysis of CSER of CAR T Therapy in Patients with Different Malignancies

**Figure 4:**
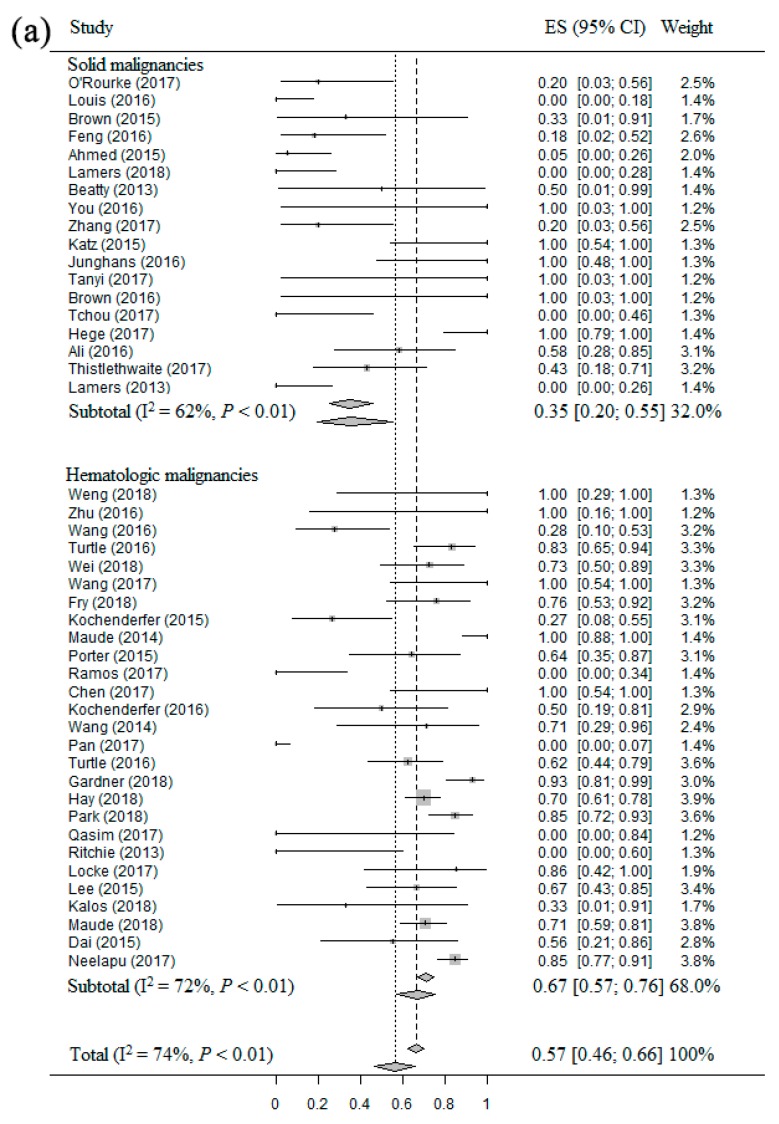

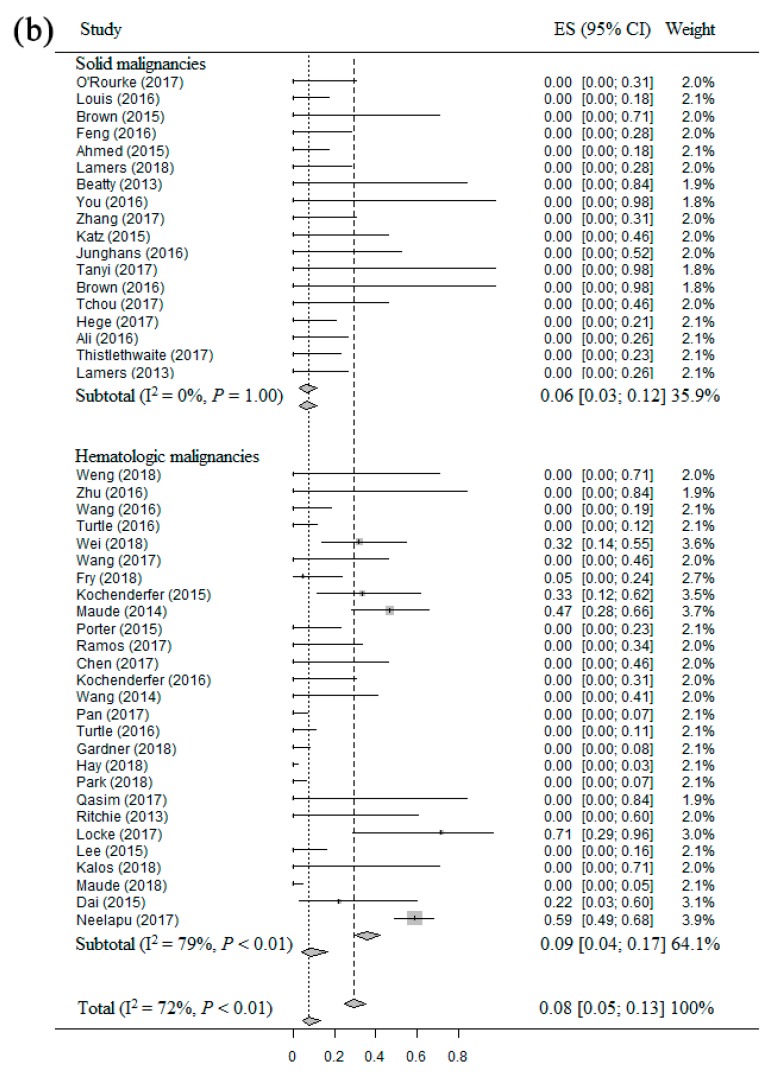

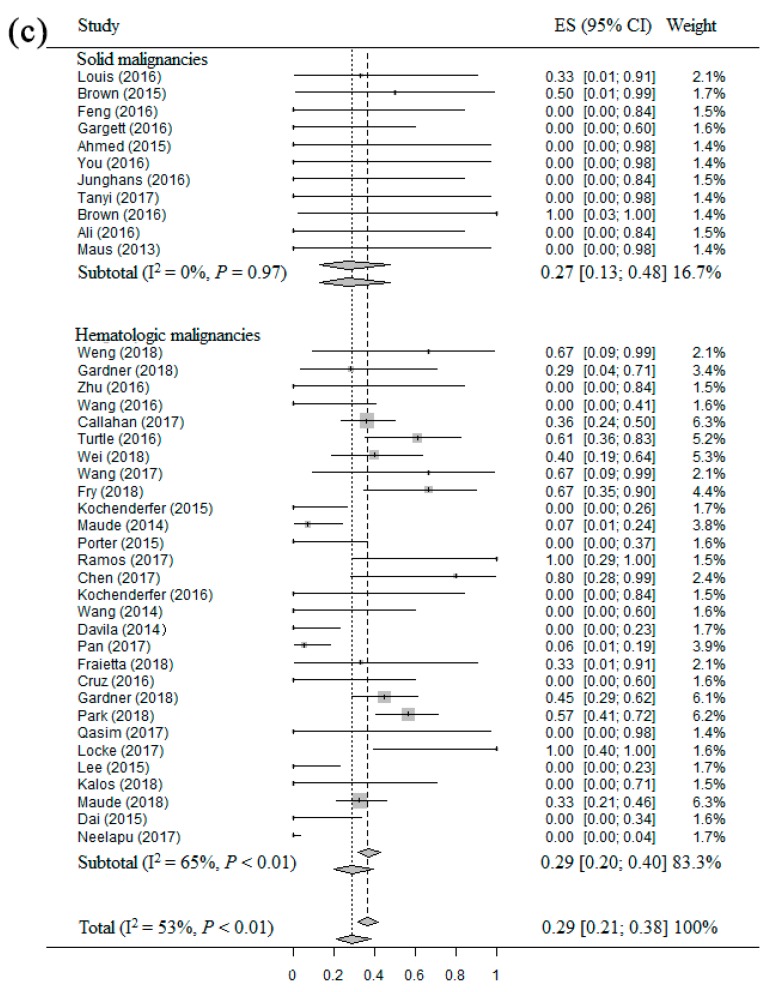
The forest plots of meta-analysis about CSER and RR: (**a**) Forest plot for CRS rate and CI in solid and hematologic malignancies patients of each study and the overall; (**b**) Forest plot for NS rate and CI in hematologic malignancies patients of each study and the overall; (**c**) Forest plot for RR and CI in solid and hematologic malignancies patients of each study and the overall.

The common side effects caused by CAR T therapy included CRS, NS and TLS. Forty-five studies [14,15,16,17,19,20,21,22,23,24,25,26,27,28,29,30,32,33,34,36,37,39,40,41,42,43,44,45,46,47,48,49,51,53,55,56,57,58,59,60,61,62,63,64,65] including 896 patients were eligible for the CSER evaluation of CAR T therapy in patients with solid and hematologic malignancies. Firstly, the overall estimate of CRS rate and 95% CI from the individual studies were 57% (95% CI: 46–66%), with a high heterogeneity (I^2^ = 74%, *p* < 0.01) (Figure 4a). Therefore, we used the random-effect model to calculate them. The CRS rate was significantly higher for patients with hematologic malignancies (67%, 95% CI: 57–76%) compared to patients with solid malignancies (35%, 95% CI: 20–55%). Secondly, the overall estimate NS rate and 95% CI from the individual studies were 8% (95% CI: 5–13%), with a high heterogeneity (I^2^ = 53%, *p* < 0.01) (Figure 4b). Thus, the random-effect model was used to calculate them. The result confirmed that the NS rate was slightly higher for patients with hematologic malignancies (9%, 95% CI: 4–17%) compared to patients with solid malignancies (6%, 95% CI: 3–12%). Beside, only one patient had TLS, thus it was not eligible for analysis in this study.

### 2.6. Meta-Analysis of RR of CAR T Therapy in Patients with Solid and Hematologic Malignancies

Forty studies [15,16,17,18,19,22,25,26,27,30,31,34,35,36,37,38,39,40,41,42,43,44,45,46,47,48,49,50,51,52,54,55,57,58,60,61,62,63,64,65], including 533 patients who achieved overall response after treatment, were eligible for the RR evaluation of CAR T therapy in patients with solid and hematologic malignancies. The overall pooled RR and 95% CI from the individual studies were 29% (95% CI: 21–38%) (Figure 4c), with a significant heterogeneity (I^2^ = 53%, *p* < 0.01). The random-effect model was used to calculate them. The subgroup analysis of solid and hematologic malignancies showed that the RR of patients with hematologic malignancies (29%, 95% CI: 20–40%) was similar to patients with solid malignancies (27%, 95% CI: 13–48%).

### 2.7. Sources of Heterogeneity

The heterogeneity analysis results showed that heterogeneity also existed in other factors, such as cell culture with the addition of IL-2. Table 3 summarized the results of meta-regression analysis.

**Figure 5:**
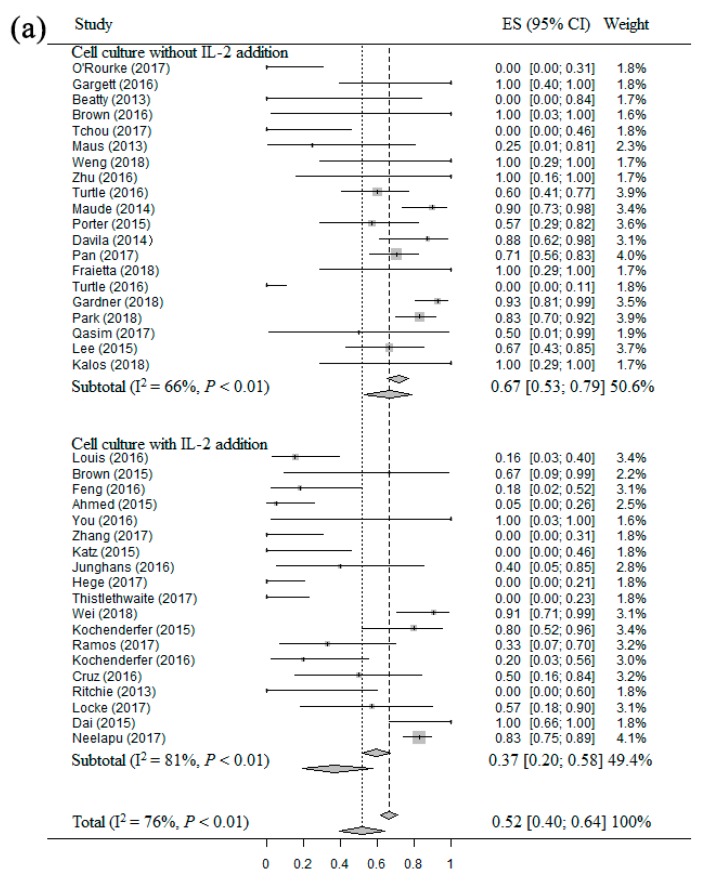

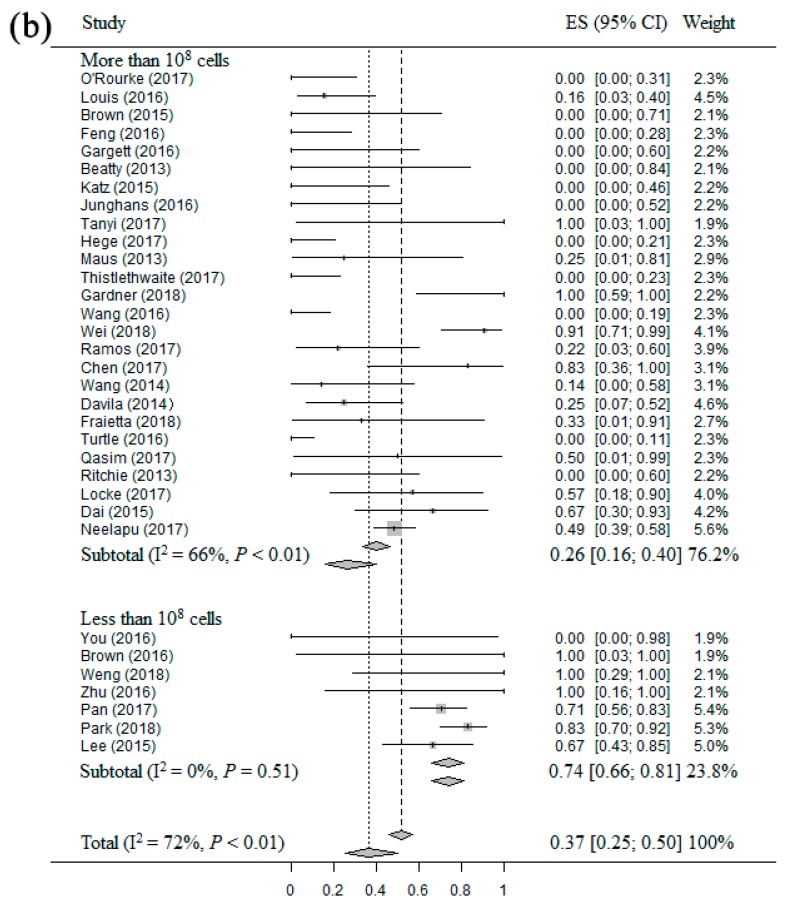
The forest plots of meta-regression analysis: (**a**) Forest plot for ORR and CI of cell culture with IL-2 addition and cell culture without IL-2 addition of each study and the overall; (**b**) Forest plot for CRR and CI of the total administration dose more than 10^8^ cells and less than 10^8^ cells of each study and the overall.

The result of meta-regression analysis between overall response and different factors showed that ORR presented a significant associations with “IL-2 addition” (*p* = 0.0176). And the result of meta-regression analysis between complete response and different factors showed that CRR had a significant associations with “total administration dose” (*p* = 0.0067). When “IL-2 addition” was entered as a variable to stratify the meta-analysis of ORR, the result showed that cell cultured without the addition of IL-2 had a higher ORR (67%, 95% CI: 53–79%) compared to cell cultured with IL-2 addition (37%, 95% CI: 20–58%) (Figure 5a). Then, when “Total administration dose” was used as a variable to stratify the meta-analysis of CRR, the result suggested that total administration dose less than 10^8^ cells had a higher CRR (74%, 95% CI: 66–81%) compared to total administration dose more than 10^8^ cells (26%, 95% CI: 16–40%) (Figure 5b).

## 3. Discussion

As one of the most promising treatments for tumors, two kinds of CAR T therapy (Tisagenlecleucel and Yescarta) had been approved by the Food and Drug Administration for patients with ALL and large B-cell lymphoma in 2017, which means that CAR T therapy had officially emerged as a regimen for tumor patients in the clinic. This decade, a lot of clinical trials regarding CAR T therapy were carried out around the world. As of April 19, 2018, the information from Clinicaltrails.gov showed that more than 270 CAR T-related studies were undergoing clinical trials in many different countries. Based on the clinical outcomes of CAR T therapy, several important factors were found. For example, if the patients with hematologic malignancies did not accept lymphodepletion before treatment with CAR T cells, they would suffer very serious CRS and even life-threatening events. However, there is still no systematic review of the efficacy and safety of CAR T therapy in patients with different tumors. Thus, this study fills this blank for the first time by using meta-analysis, subgroup analysis and meta-regression analysis.

By analyzing the ORR and CRR of CAR T therapy in patients with different tumors, we found that the response rate was significantly higher for patients with hematologic malignancies compared to patients with solid malignancies. Previous studies suggested that CAR T therapy still had many problems and challenges for patients with solid malignancies, such as antigen validation, tumor trafficking and infiltration, tumor heterogeneity, and an immunosuppressive microenvironment [27,66,67]. These problems and challenges led to poor response rate of patients with solid malignancies, and our results further confirmed it. Besides, this study analyzed the ORR and CRR of CAR T therapy in patients with different B-cell malignancies by using meta-analysis and subgroup analysis. The result confirmed that CAR T therapy had a great response rate for patients with ALL, NHL and CLL, but it had poor response rate for patients with HL.

The common side effects that caused by CAR T cells included CRS, NS and TLS. The main symptom of CRS included fever, myalgia, headache, anorexia, nausea and vomiting, renal dysfunction, coagulopathy, hypotension, capillary, leak, and pulmonary edema. The main symptom of NS included confusion, B-cell aphasia, unresponsive-ness, and seizures. And the main symptom of TLS included hyperuricemia, hyperkalemia, hyperphosphatemia, and hypocalcemia [11]. The result regarding CSER of CAR T therapy in patients with solid and hematologic malignancies suggested that the CRS rate was significantly higher for patients with hematologic malignancies compared to patients with solid malignancies, and the NS rate was slightly higher for patients with hematologic malignancies compared to patients with solid malignancies. These results confirmed that the patients with hematologic malignancies could achieve a better response rate in treatment of CAR T therapy, but they also need to take a higher risk of common side effects at the same time. More importantly, the result about RR of CAR T therapy showed that the RR of patients with hematologic malignancies was similar to patients with solid malignancies, which means that no matter the patients with solid or hematologic malignancies, they had 25–30% RR in the end. This study firstly found that different types of tumors, different CAR T protocols and even different response rate had a similar RR in the end. This finding was attributed to the inclusion of various types of tumors in the same meta-analysis.

The result of meta-regression analysis indicated that the response rate of CAR T therapy had association with “cell culture with IL-2 addition” and “total administration”. Previous study recommended that cytokines could be used in improving the expansion of first generation CARs, and it had potentially benefit for second and third generation CARs. IL-2 addition can promote the expansion of CAR T cells in vitro as a cytokine support. Zhang et al. [2] analyzed the sources of heterogeneity of CAR T therapy for patients with hematologic malignancies by using meta-regression analysis, and they found that cell cultured without IL-2 addition associated with better clinical response. However, the included studies of Zhang et al. were all published before 2015, and some studies had a same author, thus their results had some limitations. The results of this study further indicate that cell culture without IL-2 addition was associated with higher clinical response rates in patients with solid and hematologic malignancies. Then, our result also confirmed that response rate was not associated with different generation CARs, thus the need for the addition of IL-2 to culture cells remains to be studied and verified. “Total administration dose” and “persistence” in vivo were thought as two key factors for response rate and RR of CAR T therapy, respectively. Our results further confirmed that “total administration dose” had an association with response rate, but it suggested that total administration dose less than 10^8^ cells led to a better response rate, which was contrary to our long-standing perception of CAR T therapy. Furthermore, previous study [39] had confirmed that too short persistence (less than two months) could make CAR T cells no longer effective in vivo and lead to relapse. However, our result indicated that RR was not associated with “persistence”. That was the reason why we included such various CAR T protocols in the same meta-analysis, based on the meta-regression analysis of these different factors, we could identify potential factors that influenced the clinical outcomes of CAR T cells and offer a reference for the future research of CAR T therapy.

Although the subgroup analysis and meta-regression analysis were used to discuss the factors of heterogeneity in this study, this research still had some limitations. One of the most important limitations was that there are fewer patients with solid tumors, and the other limitations included ages, races, countries, etc. These limitations are inevitable and unovercomable in the present data or available data. However, the publishing time of the included studies were close (2013–2018), and the most studies were published at 2016 to 2018, then CAR T cells were almost autologous T cells. To evaluate the efficacy and safety, we tried to make a systematic analysis for CAR T therapy in all clinical studies. The publication bias main focus on index text and patient selection. Since our study included such various tumors, it was difficult to consider the identified reference standard as the best reference standard or correctly classify. CAR T therapy needs a process of production of T cells, and it resulted that some patients had not enough CAR T cells to start the treatment.

## 4. Materials and Methods

### 4.1. Search Strategy

Firstly we searched the meta-analysis regarding CAR T cells for patients with hematologic and solid malignancies. No same articles were found. Secondly, we searched studies and clinical data from the databases of PubMed, Web of Science, Wanfang and Clinicaltrials.gov. The search terms “chimeric antigen receptors”, “CAR T” and “clinic” were used in the process of search. There were no language limitations. “Full text”, “article”, “clinical research” and “completed” were used to filter articles and clinical data. Finally, the reference lists of primary studies were reviewed by two authors. The latest search happened on April 19, 2018 [68].

### 4.2. Selection Criteria for Considering Studies for This Review

All patients from included studies suffered from different hematologic or solid malignancies, and they agreed to participate in an experimental study about the efficacy and safety of treatment with CAR T cells. We excluded the following studies: (1) non-clinical research, (2) research with no clinical outcomes, (3) multiple publications of a same study, (4) graduation theses, editorials, abstracts and letters, and (5) uncompleted studies [68].

### 4.3. Initial Review of Studies

The initial database was compiled, and all duplicate articles were eliminated. We screened these citations depending on the title, abstract and the relevant studies for inclusion based on the criteria identified previously. Only after assessment of the full-text articles by two authors, the studies were finally selected for inclusion in the review. Any disagreement was resolved by discussion between two authors [68].

### 4.4. Data Abstraction

The data of initial review were recorded on a standard data extraction form by both authors independently. The name of the first author, publication year, the number of patients, types of CAR T cells, vector, original T cell sources, gene transduction method, T cell activate method, T cell culture times, CAR T cell persistence times in vivo, the types of malignancies, the method of lymphodepletion, total administration doses, clinical response and the side effects associated with CAR T therapy were all collected by two authors in each study.

### 4.5. Assessment of Study Quality and Risk of Bias

The two authors independently assessed the quality of the studies (risk and bias) by using the Quality Assessment of Diagnostic Accuracy Studies (QUADAS-2) tool [69]. According to the QUADAS-2 user guidelines [70], items were modified for this study. In domain 1 (Patient selection), the item “Was a case-control design avoided?” was omitted. In domain 2 (Index test), the items “Were the index test results interpreted without knowledge of the results of the reference standard?” and “If a threshold was used, was it pre-specified?” were substituted for the item “Was the method for determining the outcomes of patients after administration described?” In domain 3 (Reference standard), the items “Were the reference standard results interpreted without knowledge of the results of the index test?” was omitted. In domain 4 (Flow and timing), the item “Was there an appropriate interval between index test and reference standard?” was omitted, and the item “Did all patients receive the same reference standard?” and “Were all patients included in the analysis?” were replaced by the item “Did all patients accept the treatment of CAR T cells?” Based on the QUADAS-2 guidelines, researches and data were assessed for each item according to the following rating scale: high risk of bias, low risk of bias, or unclear. Any disagreement was resolved by discussion between two authors.

### 4.6. Statistical Analysis

“Meta package” is a statistical tool for meta-analysis in R software, it can be used to enhance the functionality of R software in the meta-analysis. R software has a special “Meta package” (named “Metaprop”) for rate meta-analysis. “Metaprop” realized some procedures of special binomial data, it allowed the computation of exact binomial and score test that based on CI, which means that the proportions of closing to 0 or 100% could be included from the meta-analysis, and it was used in this study. Then, the Logit transformation was used to compute the overall pooled response rate, CSER and RR.

Cochran’s Q test and Higgins’ I^2^ statistics were used to make homogeneity test for eligible studies. A *p*-value ≤ 0.1 and/or I^2^ ≥ 50% indicated significant heterogeneity, the data should be calculated by the random-effect model. Accordingly A *p*-value > 0.1 and/or I^2^ < 50% indicated significant homogeneity, the data should be calculated by fixed-effect model. [68] The subgroup analysis of hematologic and solid malignancies was used in all result of meta-analysis. Potential heterogeneity factors of each analysis, including “Generation of CARs”, “Vector”, “Cell culture time”, “Transfection method”, “IL-2 addition”, “Persistence”, “Lymphodepletion” and “Total administration dose”, were assessed by meta-regression analysis based on the Cox proportional hazards regression model. All analysis were performed by using Review Manager 5.3 (The Cochrane Collaboration, Copenhagen, Denmark), R software 3.5.0 (R Foundation for Statistical Computing, Vienna, Austria) and SPSS Statistics 22 (IBM, Armonk, NY, USA).

## 5. Conclusions

This study used meta-analysis to further confirm that CAR T therapy had a higher ORR and CRR in patients with hematologic malignancies compared to patients with solid malignancies, especially in patients with ALL, NHL and CLL. More importantly, this study confirmed that the patients with hematologic malignancies had a higher CSER, and patients with hematologic and solid malignancies had a similar RR in the end. The meta-regression analysis results suggest that cell cultured without IL-2 addition and total administration less than 10^8^ cells were recommended for manufacture of CAR T cells and clinical treatment, respectively. These results may make CAR T therapy becoming cheaper, because the cost of IL-2 could be saved, and the cost of CAR T cells amplification could be appropriately reduced. The number of patients who were unable to be treated due to not having enough CAR T cells would also be decreased. We believe that CAR T therapy could play an increasingly important role in tumor treatment in the future.

## Figures and Tables

**Table 1:** Basic patient characteristics of the studies included in the meta-analysis and the results of methodological quality assessment using the QUADAS-2 tool.

Author, Year (Ref.)	Age * (Years Old)	Sex	QUADAS				
			1	2	3	4	5
O’Rourke et al. 2017 [14]	59.5 (45–76)	5M/5F					
Louis et al. 2016 [15]	7 (3–20)	10M/9F					
Brown et al. 2015 [16]	50 (36–57)	1M/2F					
Feng et al. 2016 [17]	58 (40–66)	5M/6F					
Gargett et al. 2016 [18]	NS	NS					
Ahmed et al. 2015 [19]	17 (7–29)	9M/10F					
Lamers et al. 2018 [20]	NS	NS					
Beatty et al. 2013 [21]	78 (81 and 75)	2M					
You et al. 2016 [22]	NS	NS					
Zhang et al. 2017 [23]	58 (48–67)	7M/3F					
Katz et al. 2015 [24]	57 (51–66)	6M/2F					
Junghans et al. 2016 [25]	61(51–75)	5M					
Tanyi et al. 2017 [26]	52	1F					
Brown et al. 2016 [27]	50	1M					
Tchou et al. 2017 [28]	55.3 (44–64)	6F					
Hege et al. 2017 [29]	56	9M/5F					
Ali et al. 2016 [30]	NS	NS					
Maus et al. 2013 [31]	NS	NS					
Thistlethwaite et al. 2017 [32]	47.4 (36–66)	8M/6F					
Lamers et al. 2013 [33]	60.8 (46–74)	8M/4F					
Weng et al. 2018 [34]	23.3 (16–34)	2M/1F					
Gardner et al. 2018 [35]	NS	NS					
Zhu et al. 2016 [36]	34 (29–39)	1M/1F					
Wang et al. 2016 [37]	33 (13–77)	13M/5F					
Callahan et al. 2017 [38]	(1–14)	NS					
Turtle et al. 2016 [39]	40 (20–73)	NS					
Wei et al. 2018 [40]	35.8 (8–57)	10M/13F					
Wang et al. 2017 [41]	7 (5–9)	5M/1F					
Fry et al. 2018 [42]	19 (7–30)	13M/8F					
Kochenderfer et al. 2015 [43]	51.7(30–68)	8M/7F					
Maude et al. 2014 [44]	14 (5–60)	18M/12F					
Porter et al. 2015 [45]	66 (51–78)	12M/2F					
Ramos et al. 2017 [46]	34.7 (20–65)	6M/3F					
Chen et al. 2017 [47]	26.5 (8–44)	1M/5F					
Kochenderfer et al. 2016 [48]	52.4 (44–66)	8M/2F					
Wang et al. 2014 [49]	62.4 (37–85)	6M/1F					
Davila et al. 2014 [50]	50(18–59)	12M/4F					
Pan et al. 2017 [51]	13.3 (2–68)	32M/19F					
Fraietta et al. 2018 [52]	62 (57–68)	3M					
Turtle et al. 2016 [53]	57 (22–70)	27M/5F					
Cruz et al. 2016 [54]	51 (9–59)	5M/3F					
Gardner et al. 2018 [55]	12.2 (1–25)	23M/22F					
Hay et al. 2018 [56]	54 (20–73)	93M/40F					
Park et al. 2018 [57]	44 (23–74)	NS					
Qasim et al. 2017 [58]	13.5 † (11 † and 16 †)	2F					
Ritchie et al. 2013 [59]	70.6 (64–78)	1M/3F					
Locke et al. 2017 [60]	52.3 (29–69)	5M/2F					
Lee et al. 2015 [61]	14.7 (5–27)	14M/7F					
Kalos et al. 2018 [62]	68.7 (64–77)	3M					
Maude et al. 2018 [63]	11 (3–23)	NS					
Dai et al. 2015 [64]	38.9 (15–65)	4M/5F					
Neelapu et al. 2017 [65]	58 (23–76)	68M/43F					

Notes: * mean and range, † month, M: male, F: female, NS: not specified. Items of modified QUADAS-2 tool used in this study: (1) Was a consecutive or random sample of patients enrolled? (2) Did the study avoid inappropriate exclusions? (3) Was the method for determining the outcomes of patients after administration described? (4) Is the reference standard likely to correctly classify the target condition? (5) Did all patients accept the treatment of CAR T cells? According to the QUADAS-2 manual, each item was assessed as “yes”, “no” or “unclear”:
    YesNoUnclear

**Table 2:** Clinical trial characteristics.

No ^a^	CAR T Type	Vector	T Cell Origin	Cell Culture	Transfection Method	T Cell Treatment	CAR T Cell Persistence	Diagnosis	Lympho-depletion	Dose ^b^	Responses	Side Effects ^d^	Ref
10	CART-EGFRvIII	CD8+4-1BB+CD3	Autologous	10 days	Lentivirus	CD3/28 beads	<30 days	GBM	Temozolomide, radiation	5 × 10^8^ cells	2 PD, 1 SD, 7 Died	Seizure, headache, weakness, cerebral edema	[14]
19	GD2-specific CAR T	Zeta5+Zeta6	Autologous	6 weeks	Retroviral	OKT3+IL-2	96–192 weeks	NB	No ^c^	2 × 10^7^ cells/m^2^, 5 × 10^7^ cells/m^2^, 1 × 10^8^ cells/m^2^	3 CR, 1 PR, 8 NED, 4 PD, 1 SD, 2 Tumor necrosis, 1 Relapse	Localized pain	[15]
3	IL13Rα2-specific CD8 + CAR T	IL-13+ CD4+CD3	Autologous	14 days	Electroporation	OKT3+IL-2+irradiated feeders	14 weeks	GBM	NI	9.6 × 10^8^ cells, 10.6 × 10^8^ cells, 10.6 × 10^8^ cells	2 PR, 1 Relapse	Headaches, neurologic-shuffling gait	[16]
11	CAR T- EGFR	CD8+4-1BB+CD3	Autologous	10 to 13 days	Lentiviral	CD3+GT-T551+IL2	>4 weeks	NSCLC	CY, pemetrexed, CDDP, docetaxel	9.7 × 10^6^ cells/kg	2 PR, 4 PD, 5 SD	Rash, acne, dry skin, nausea, vomiting dyspnea, hypotension, serum amylase elevation, serum lipase elevation	[17]
4	GD2-specific CAR T	CD28+OX40	Autologous	13–17 days	Retroviral	CD3/28 beads	42 days	Metastatic melanoma	FLU, CY	1 × 10^7^ cells/m^2^, 2 × 10^7^ cells/m^2^	4 PR	NI	[18]
19	HER2-CAR T	HER2+CD28	Autologous	10–21 days	Retroviral particles	CD3/CD3+CD8+IL2	<18 months	OS, EWS, PNET, DSRCT	Chemotherapy radiation	1 × 10^4^ cells/m^2^, 1 × 10^8^ cells/m^2^	1 PR, 12 PD, 4 SD, 2 NE	Fever	[19]
11	CAIX-CAR T	SFG-derived	Autologous	5 weeks	Retroviral	IL-2/anti-CAIX mab cg250	<29 days	RCC	No ^c^	3 × 10^8^–2.1×10^9^ IU/m^2^	NI	Liver toxicities	[20]
2	CART-meso	4-1BB	Autologous	8–12 days	Electroporation	CD3/28 beads	1 weeks or 13 days	MPM, PDA	Chemotherapy	4.4 × 10^9^ cells, 3 × 10^8^ cells/m^2^	2 SD	Cardiac arrest, respiratory failure, disseminated intravenous coagulation, CRS, jejunal obstruction, abdominal pain, lymphocytosis	[21]
1	anti-MUC1 CAR T	CD28+4-1BB+CD3z	Autologous	12 days	Lentiviral	IL-2+IL-21	NI	SVL	No ^c^	5 × 10^5^ cells	1 PR	Mild headache, muscle pain, nasal congestion, mild abdominal bloating discomfort	[22]
10	CEA CAR T	CD28-CD28+CD3	Autologous	2 weeks	Lentiviral	IL-2	6 weeks	mCRC	CY+FLU	2.5 × 10^7^–1.5 × 10^10^ cells	2 PD, 7 SD, 1 NE	Fever	[23]
6	CEA CAR T	CD28+CD3	Autologous	10–14 days	Retroviral	CD3+OKT3+IL-2	1 months	Liver metastases	NI	1.11 × 10^10^ cells, 3 × 10^10^ cells	5 PD, 1 SD	Fever, mylagias, abdomnal pain, nausea, emesis, tachycardia, abdominal wall muscle spasm, ALT↑, AST↑, alk phos↑, ascites, edema, thrombo-cytopenia, leukopenia, dyspnea, pleural effusion, anorexia, rash, subscapular liver hematoma, eosinophilia, chills, bilirubin up, diarrhea, dehydration, colitis	[24]
5	PSMA designer CAR T	NI	Autologous	20 days	Retrovirus	CD3+IL-2	NI	PCa	Non-myeloablative chemo-therapy+IL-2	1 × 10^9^ cells, 1 × 10^10^ cells	2 PR, 1 Minor response, 2 NR	Fever, amemia, hypocalcemia, hypophosphatemia, appendicitis	[25]
1	CART-meso	4-1BB+ TCR-zeta	Autologous	10 days	Lentiviral	NI	26 days	Ovarian cancer	No ^c^	3 × 10^7^ cells/m^2^	1 CR	Fever, high volume pleural fluid	[26]
1	IL13Rα2-specific CD8 + CAR T	CD4+4-1BB+CD3	Autologous	18 days	Lentiviral	CD3/CD28 beads	At least 7 days	GBM	No ^c^	5.2 × 10^7^ cells	1 CR, 1 Relapse	Headaches, generalized fatigue, myalgia, olfactory auras	[27]
6	mRNA c-Met-CAR T	4-1BB+TCR	Autologous	21 days	Electroporation	CD3/28 beads	7 days	Breast cancer	No ^c^	3 × 10^7^ cells, 3 × 10^8^ cells	2 PD, 1 SD, 3 Died	No side effect	[28]
16	CART72	Igg1+CH3+CD4+CD3	Autologous	10–17 days	Retroviral	OKT3+CD28+IL-2 beads	Most ≤14 weeks and one 48 weeks	CRC	No ^c^	3 × 10^9^–4 × 10^9^ cells	3 PD	Chills, fever, dizziness, paresthesia, headache, tachycardia, myalgia, hypoxia, low grade CRS, IFN toxicity	[29]
12	CAR-BCMA T	CD28+CD3	Autologous	9 days	Retroviral	NI	<14 weeks	MM	CY+FLU	3 × 10^5^–9 × 10^6^ cells/kg	1 Stringent CR, 1 PR, 8 SD, 2 VGPR	Hypophosphatemia, anemia, nausea, headache, hypocalcemia, upper respiratory infection, fever, atrial fibrillation, thromboembolic event, rash, dyspnea, delirium, epistaxis	[30]
4	CART-meso	4-1BB+TCR	Autologous	10 days	Electroporation	CD3/28 beads	NI	PAC, MPM	No ^c^	1.1 × 10^9^ cells	1 CR, 1 PD, 2 SD, 1 Died	NI	[31]
14	CEA CAR T	CD3+pmp71	Autologous	9 days	Retroviral vector	OKT3+IL-2	<14 days	Adeno-carcinoma, PMP	FLU+CY	1 × 10^9^–5 × 10^10^ cells	7 SD, 7 PD	Pelvic pain, neutropaenia, general deterioration, hypocalcaemia, leukopenia, hypophosphataemia, lymphopenia, left side pain, abdominal pain, anaemia, thrombocytopenia, vomiting, hypotension, hyperbilirubinaemia, hyoialbuminaemia, epistaxis, hyponatraemia, intermittent pyrexia, haematemesis, jaundice, neutropenic sepsis, intermittent anaemia, intermittent increased respiratory rate	[32]
12	CAIX-CAR T	G250-CD4	Autologous	15 days	Retroviral	CD3+OKT, CD3+CD28, HPA	<37 days	RCC	No ^c^	6 × 10^8^–4 × 10^9^ cells	NI	Liver enzyme disturbances	[33]
3	1928zT2 CAR T	CD28+CD3+TIR	Autologous	14 days	Lentiviral	CD3/CD28 beads	<92 days	B-ALL	FLU+CY	5 × 10^4^–1 × 10^6^ cells/kg	3 CR, 2 Relapse	Fever, left breast pain, transient neutropenia, bone pain, IL-6↑, PCT↓, IL-6↑and↓	[34]
7	CD19 CAR T	NI	Autologous	NI	NI	NI	22 or 30 days	B-ALL	Chemotherapy	2 × 10^6^ cells/kg, 1 × 10^7^ cells/kg	7 CR, 2 Relapse	NI	[35]
2	CD19 CAR T	4-1BB+CD3	Autologous	7 days	Lentivirus	CD3/CD28 beads	4 weeks	B-ALL	FLU+CY	1.19 × 10^6^ cells/kg, 1.0 × 10^6^ cells/kg	2 CR	Fever, hypotension, cytokine levels↑, CRP, ferritin	[36]
18	CAR T30	4-1BB+CD3	Autologous	12 days	Lentiviral	NI	3 months	HL	FLU+CY	1.56 × 10^7^ cells/kg	7 PR, 6 SD	Febrile syndrome, vomiting, urticarial-like rash, breathlessness, psychiatric abnormalities, joint swelling, dizziness, pneumonitis, ALT↑, AST↑, γ-GGT↑, triglyceride, abnormalities of aminotransferase, left ventricular systolic function	[37]
59	CD19 CAR T	NI	Autologous	3 weeks	Lentiviral and retroviral	NI	NI	B-ALL	Chemotherapy	NI	55 CR, 20 Relapse	NI	[38]
30	CD19 CAR T	Igg4+CD28+4-1BB+CD3	Autologous	15–20 days	Lentiviral	CD3/CD28 beads	90 days	B-ALL	CE, CY, CY+FLU5, CY+FLU3	2 × 10^5^ cells/kg, 2 × 10^6^ cells/kg, 2 × 10^7^ cells/kg	5 Died in CR, 13 Alive in CR, 2 MRD, 1 NE, 11 Relapse	CRS, severe neurotoxicity, generalized eizures, transient disseminated intravascular coagulation	[39]
22	CD19 CAR T	CD8+4-1BB+CD3	Autologous	11 days	Lentiviral	IL-2	NI	B-ALL	FLU+CY	3.0 × 10^7^–10 × 10^7^ cells/kg	20 CR, 2 SD, 1 Died, 8 Relapse	Blood bilirubin increased, hypotension, APTT prolonged, fibrinogen decreased, diarrhea, vomit, CRS, aspartate aminotransferase increased, alanine aminotransferase increased, hypoalbuminemia, neurologic event, infusion reaction	[40]
6	CD19 CAR T	NI	Autologous	NI	NI	NI	<21 days	B-ALL	CY+BU+FLU	1.2 × 10^6^–8.5 × 10^6^ cells/kg	3 CR, 1 Died, 2 NR, 2 Relapse	CRS	[41]
21	CD22-CAR T	CD8+CD3+4-1BB	Autologous	14 days	Lentiviral	NI	2 months	B-ALL	FLU+CY	3 × 10^5^ cells/kg 1 × 10^6^ cells/kg 3 × 10^6^ cells/kg	12 CR, 9 MRD, 8 Relapse	CRS, self-limited, noninfectious diarrhea, hypoxia, transient visual hallucinations, mild unresponsiveness, mild disorientation, mild–moderate pain	[42]
15	CD19 CAR T	CD28+CD3	Autologous	10 days	Gamma-retroviral	CD3+OKT3+IL2	<75 days	SMZL, PMBCL, CLL, DLBCL, NHL	FLU+CY	1 × 10^6^–5 × 10^6^ cells/kg	8 CR, 4 PR, 1 SD, 2 Died	Hypotension, serum interferon gamma and/or IL-6↑, neurologic abnormalities, intermittent aphasia, confusion, severe generalized myoclonus,	[43]
30	CD19 CAR T	CD3+CD28+4-1BB	Autologous	8–12 days	Lentiviral	CD3/CD28 beads	2 years	B-ALL	CY+VP, FLU+CY, Clofarabine, CVDA-B, CVDA-A	7.6 × 10^5^–2.06 × 10^7^ cells/kg	27 CR, 3 NR, 2 Relapse	CRS, neurologic toxic effects, delayed encephalopathy, seizures	[44]
14	CD19 CAR T	CD3+4-1BB	Autologous	10–12 days	Lentiviral	CD3/CD28 beads	4 years	CLL	Bendamustine, FLU+CY, Pentostatin+CY	1.4 × 10^7^–11 × 10^8^ cells	4 CR, 4 PR, 6 NR	Tumor lysis syndrome, CRS	[45]
9	CART-30	Igg1.CH2-CH3+CD28	Autologous	1–3 months	Retroviral	OKT3/CD3+CD28+IL2	>6 months	HL	No ^c^	2 × 10^7^–2 × 10^8^ cells/m^2^	2 CR, 1 CCR, 3 SD, 3 NR, 7 Relapse	No side effects	[46]
6	CD19 CAR T	CD28/CD27+caspase 9	Autologous	5–15 days	Lentiviral	NI	<2 months	B-ALL	CY+FLU	3.8 × 10^7^–4.1 × 10^8^ cells/kg	5 MRD-negative CR, 1 NR, 4 Relapse	Agvhd, fever, dysfunctional blood coagulation, rash, diarrhoea, hypotension, hypoxia	[47]
10	CD19 CAR T	CD28+CD3	Autologous	8 days	Gamma-retroviruses	OKT3+IL-2	About 1 month	CLL, DLBCL, MCL	No ^c^	1 × 10^6^–5.9 × 10^7^ cells/kg	2 PD, 6 SD, 2 PR	Tumor lysis syndrome, fatigue, cardiac, ventricular dysfunction, fever, tachycardia, troponin increase, anemia, neutropenia, pneumonitis, hypoxia, dyspnea, hypophosphatemia, hypotension, headache	[48]
7	anti-CD20 CART	CD20+4-1BB+CD3	Autologous	13 days	Lentiviral	NI	10 weeks	DLBCL	CY, VCR, VP, DEX, ADM, MPN, CBP, ARA-C	4.1 × 10^6^ –1.46 × 10^7^ cells/kg	1 CR, 3 PR, 2 PD, 1 NE	CRS, alimentary tract hemorrhage, sudden tumor lysis syndrome, capillary leak syndrome, acute alimentary tract hemorrhage, lung dysfunction, glossopharyngeal mucusdamage, serous cavity effusion	[49]
16	19-28z CAR T	CD28+CD3	Autologous	14 days	Lentiviral	CD3/CD28 beads	2–3 months	B-ALL	CY	3 × 10^6^ cells/kg	10 CR, 4 CRi, 2 NR	NI	[50]
51	CD19 CAR T	CD8+4-1BB+CD3	Autologous	7–8 days	Lentiviral	CD3/CD28 beads	<60 days	B-ALL	CY+FLU	5 × 10^3^–1.4 × 10^7^ cells/kg, 1 × 10^5^ cells/kg	36 CR, 9 MRD, 3 NR, 3 Died, 2 Relapse	Seizure, short time coma, severe coagulation disorders, intracranial hemorrhage, heart failure	[51]
3	CD19 CAR T	CD8+4-1BB+CD3	Autologous	NI	Lentiviral	CD3/CD28 beads	NI	CLL	NI	1 × 10^8^–1 × 10^9^ cells	1 CR, 2 PR, 1 Relapse	NI	[52]
32	CD19 CAR T	Igg4+CD28+4-1BB+CD3	Autologous	15 days	Lentiviral	CD3/CD28 beads	NI	NHL	CY+FLU, CY, CY+VP	8.8 × 10^6^ cells/kg 7.0 × 10^6^ cells/kg	1 Relapse	Concentrations of serum cytokines↑, fever, and/or hypotension consistent with CRS, severe neurotoxicity, encephalopathy alone, tremor, speech disturbance	[53]
8	CD19 CAR T	CD28	Autologous	5–6 weeks	Retroviral	Ad5f35pp65+IL-2	<12 weeks	CLL, B-ALL	No ^c^	1.9 × 10^7^-1.13 × 10^8^ cells	1 CR, 2 CCR, 1 PR, 3 PD, 1 SD,	NI	[54]
43	CD19 CAR T	4-1BB	Autologous	20–22 days	Lentiviral	CD3/CD28 beads	2–28 months	B-ALL	FLU+CY, CY	5 × 10^5^–1 × 10^7^ cells/kg	40 MRD-CR, 3 NC, 18 Relapse	CRS, sCRS, neurotoxicity, neurotoxicity	[55]
133	CD19 CAR T	Igg4+CD28+4-1BB+CD3	Autologous	15 days	Lentiviral	CD3/CD28 beads	NI	B-ALL, CLL, NHL	FLU+CY	2 × 10^5^-2 × 10^6^ cells/kg	NI	CRS, fever	[56]
53	CD19 CAR T	CD28+CD3	Autologous	NI	Retrovirus	CD3/CD28 beads	7–138 days	B-ALL	Chemotherapy	1 × 10^6^ cells/kg	44 CR, 8 NR, 1 Died, 25 Relapse	CRS, sCRS, neurotoxic effects	[57]
2	CD19 CAR T	4g7+4-1BB+CD3	Autologous	18 days	Lentiviral	CD3/CD28 beads	9 weeks	B-ALL	FLU+CY, FLU+CY+, alemtuzumab	4.6 × 10^6^ cells/kg, 4.0 × 10^6^ cells/kg	1 CR, 1 Remains clinically well	Transient erythematous rash	[58]
4	LeY CAR T	CD8+CD28+CD3	Autologous	12 days	Retroviral	OKT-3+IL-2	10 months	AML	FLU_+_CY	5 × 10^8^–1.3 × 10^9^ cells	1 Cytogenetic remission, 3 Protracted remission, 1 Relapse	Transient neutropenia	[59]
7	CD19 CAR T	CD3+CD28	Autologous	8–9 days	Retroviral	CD3+IL-2	12 months	DLBCL	FLU+CY	2 × 10^6^ cells/kg	4 CR, 2 NR, 4 Relapse	CRS, neurotoxicity, febrile neutropenia, encephalopathy, neutropenia, anemia, hypoxia, somnolence, oral herpes, thrombocytopenia, acute kidney injury, agitation, ascites, increased aspartate aminotransferase. cardiac failure, delirium, fatigue, hemorrhage intracranial, hypocalcemia, hyponatremia, metabolic acidosis, hypo-phosphatemia, hypo-tension, pseudomonal sepsis, pyrexia, restlessness, tremor, urinary tract infection	[60]
21	CD19 CAR T	MSVG-FMC63-28z	Autologous	NI	Retroviral	CD3/CD28 beads	Most <28 days	B-ALL	FLU+CY	1 × 10^6^ cells/kg	13 CR, 1 CRi, 4 PD, 3 SD	Acute kidney injury, cardiac arrest, CRS, qtc prolongation, febrile neutropenia, fever, hypertension, hypotension, hypoxia, dysfunction, multi-organ failure, pulmonary oedema, respiratory failure, prolonged activated partial thromboplastin time, anaemia, ALT↑, AST↓, blood bilirubin↑, cpk↑, hyperglycaemia, hypo-kalaemia, hyponatraemia, hypophosphataemia, ataxia, dysphasia, headache, tremor	[61]
3	CD19 CAR T	4-1BB+CD3	Autologous	4 weeks	Lentiviral	CD3/CD28 beads	>6 months	CLL	Bendamustine, bendamustine+rituximab, pentostatin+CY	1.46 × 10^5^–1.1 × 10^9^ cells/kg	2 CR, 1 PR	Transient febrile reaction, rigors, dyspnea, transient cardiac dysfunction, transient hypotension	[62]
75	CD19 CAR T	4-1BB+CD3	Autologous	NI	Lentiviral	NI	20–617 days	CLL	Chemotherapy	2 × 10^5^–5.4 × 10^6^ cells/kg	45 CR, 16 CRi, 20 Relapse	CRS, hypotension, lymphocyte count↓, hypoxia, increase in blood bilirubin, increase in aspartate aminotransferase, pyrexia, acute kidney injury, hypophosphatemia, hypokalemia, pulmonary edema, thrombocytopenia, encephalopathy, alanine aminotransferase↑, fluid overload	[63]
9	CD19 CAR T	4-1BB+CD3	Autologous and donor-derived	10–12 days	Lentiviral	OKT3+IL-2	3 months	B-ALL	NI	3 × 10^6^–1 × 10^7^ cells/kg	6 CR, 3 PR	Chills, fever, CRS, neurological symptoms, gvhd, acute capillary leaking, syndrome, lung and pancreas injuries, tumor lysis syndrome, oral and genital mucosa ulcers	[64]
111	CD19 CAR T	CD8+CD28+4-1BB	Autologous	20–24	Retroviral	CD3+OKT3+IL-2	>30 days, <90 days	DLBCL, PMBCL or TFL	FLU+CY	2 × 10^6^ cells/kg	54 CR, 28 PR, 11 SD, 5 PD, 2 NE	Pyrexia, neutropenia, anemia, hypotension, thrombocytopenia, nausea, fatigue, decreased appetite, headache, diarrhea, hypoalbuminemia, hypocalcemia, chills, tachycardia, febrile neutropenia, encephalopathy, thrombocytopenia, vomiting, hypokalemia, hyponatremia, constipation, white-cell count↓, CRS, neurologic event	[65]

Note: No ^a^: number of included patient, Dose ^b^: total dose of infused CAR T cells, No ^c^: not accept lymphodepletion, Side Effect ^d^: Side effects associated with CAR T cells, NI: not information, NB: neuroblastoma, OS: osteosarcomas, PNET: primitive neuroectodermal tumer, SVL: seminal vesicle cancer, PCa: prostate cancer, MM: multiple myeloma, RCC: renal cell carcinoma, PDA: pancreatic cancer, mCRC: metastatic colorectal cancer, PMP: pseudomyxoma peritonei, PAC: pancreatic adenocarcinoma, CLL: chronic lymphocytic leukemia, CY: cyclophosphamide, CDDP: cisplatin, FLU: fludarabine, CE: cyclophosphamide+etoposide, BU: busulfan, VP: etoposide, CVDA-B: methotrexate+cytarabine, CVDA-A: cyclophosphamide+vincristine+adriamycin, VCR: vincristine, DEX: dexamethasone, ADM: doxorubicin, MPN: methylprednisolone, CBP: carboplatin, ARA-C: cytosine arabinoside, SD: stable disease, PD: progressive disease, NED: indicates no evidence of disease, CR: complete response, PR: partial response, NE: not evaluated, VGPR: very good partial response, MRD: minimal residual disease, NR: not response, CCR: continued complete response, CRi: complete response with incomplete count recovery, ↑: increase, ↓: decrease, CRS: cytokine release syndrome, ALT: alanine aminotransferase, AST: aspartate aminotransferase, PCT: procalcitonin, TFL: transformed follicular lymphoma.

**Table 3:** The result of meta-regression analysis about the association between the characteristic of cohorts and the difference rates included in this studies.

Variable	*p*				
	Overall Response	Complete Response	CRS	NS	Relapse
Generation of CAR	0.2949	0.4523	0.5202	-	0.9884
Vector	0.9289	0.8990	0.0748	0.2253	0.2467
Cell culture time	0.8076	0.7688	0.1466	0.2253	0.8455
Transfection method	0.5534	0.1119	0.4349	0.2253	0.6129
IL-2 addition	0.0176	0.1119	0.7218	0.2253	0.6129
Persistence	0.4836	0.5633	0.2279	-	0.8455
Lymphodepletion	0.9053	0.9938	0.3513	-	0.6129
Total administration dose	0.2022	0.0067	0.1466	0.1573	0.6129

## References

[1] Maus M.V., Grupp S.A., Porter D.L., June C.H. (2014). Antibody-modified T cells: CARs take the front seat for hematologic malignancies. Blood.

[2] Zhang T., Cao L., Xie J., Shi N., Zhang Z., Luo Z., Yue D., Zhang Z., Wang L., Han W. (2015). Efficiency of CD19 chimeric antigen receptor-modified T cells for treatment of B cell malignancies in phase I clinical trials: A meta-analysis. Oncotarget.

[3] Topalian S.L., Wolchok J.D., Chan T.A., Mellman I., Palucka K., Banchereau J., Rosenberg S.A., Dane Wittrup K. (2015). Immunotherapy: The path to win the war on cancer?. Cell.

[4] Tang X.J., Sun X.Y., Huang K.M., Zhang L., Yang Z.S., Zou D.D., Wang B., Warnock G.L., Dai L.J., Luo J. (2015). Therapeutic potential of CAR-T cell-derived exosomes: A cell-free modality for targeted cancer therapy. Oncotarget.

[5] Magee M.S., Snook A.E. (2014). Challenges to chimeric antigen receptor (CAR)-T cell therapy for cancer. Discov. Med..

[6] Kochenderfer J.N., Wilson W.H., Janik J.E., Dudley M.E., Stetler-Stevenson M., Feldman S.A., Maric I., Raffeld M., Nathan D.A., Lanier B.J. (2010). Eradication of B-lineage cells and regression of lymphoma in a patient treated with autologous T cells genetically engineered to recognize CD19. Blood.

[7] Carpenito C., Milone M.C., Hassan R., Simonet J.C., Lakhal M., Suhoski M.M., Varela-Rohena A., Haines K.M., Heitjan D.F., Albelda S.M. (2009). Control of large, established tumor xenografts with genetically retargeted human T cells containing CD28 and CD137 domains. Proc. Natl. Acad. Sci. USA.

[8] Van der Stegen S.J., Hamieh M., Sadelain M. (2015). The pharmacology of second-generation chimeric antigen receptors. Nat. Rev. Drug Discov..

[9] Allegra A., Innao V., Gerace D., Vaddinelli D., Musolino C. (2016). Adoptive immunotherapy for hematological malignancies: Current status and new insights in chimeric antigen receptor T cells. Blood Cells Mol. Dis..

[10] Yeku O.O., Brentjens R.J. (2016). Armored CAR T-cells: Utilizing cytokines and pro-inflammatory ligands to enhance CAR T-cell anti-tumour efficacy. Biochem. Soc. Trans..

[11] Smith L., Venella K. (2017). Cytokine Release Syndrome: Inpatient Care for Side Effects of CAR T-Cell Therapy. Clin. J. Oncol. Nurs..

[12] Zhang Q., Zhang Z., Peng M., Fu S., Xue Z., Zhang R. (2016). CAR-T cell therapy in gastrointestinal tumors and hepatic carcinoma: From bench to bedside. Oncoimmunology.

[13] Fesnak A., Lin C., Siegel D.L., Maus M.V. (2016). CAR-T Cell Therapies From the Transfusion Medicine Perspective. Transfus. Med. Rev..

[14] O’Rourke D.M., Nasrallah M.P., Desai A., Melenhorst J.J., Mansfield K., Morrissette J.J.D., Martinez-Lage M., Brem S., Maloney E., Shen A. (2017). A single dose of peripherally infused EGFRvIII-directed CAR T cells mediates antigen loss and induces adaptive resistance in patients with recurrent glioblastoma. Sci. Transl. Med..

[15] Louis C.U., Savoldo B., Dotti G., Pule M., Yvon E., Myers G.D., Rossig C., Russell H.V., Diouf O., Liu E. (2011). Antitumor activity and long-term fate of chimeric antigen receptor-positive T cells in patients with neuroblastoma. Blood.

[16] Brown C.E., Badie B., Barish M.E., Weng L., Ostberg J.R., Chang W.C., Naranjo A., Starr R., Wagner J., Wright C. (2015). Bioactivity and Safety of IL13Rα2-Redirected Chimeric Antigen Receptor CD8+ T Cells in Patients with Recurrent Glioblastoma. Clin. Cancer Res..

[17] Feng K., Guo Y., Dai H., Wang Y., Li X., Jia H., Han W. (2016). Chimeric antigen receptor-modified T cells for the immunotherapy of patients with EGFR-expressing advanced relapsed/refractory non-small cell lung cancer. Sci. China Life Sci..

[18] Gargett T., Yu W., Dotti G., Yvon E.S., Christo S.N., Hayball J.D., Lewis I.D., Brenner M.K., Brown M.P. (2016). GD2-specific CAR T Cells Undergo Potent Activation and Deletion Following Antigen Encounter but can be Protected From Activation-induced Cell Death by PD-1 Blockade. Mol. Ther..

[19] Ahmed N., Brawley V.S., Hegde M., Robertson C., Ghazi A., Gerken C., Liu E., Dakhova O., Ashoori A., Corder A. (2015). Human Epidermal Growth Factor Receptor 2 (HER2) -Specific Chimeric Antigen Receptor-Modified T Cells for the Immunotherapy of HER2-Positive Sarcoma. J. Clin. Oncol..

[20] Lamers C.H., Willemsen R., van Elzakker P., van Steenbergen-Langeveld S., Broertjes M., Oosterwijk-Wakka J., Oosterwijk E., Sleijfer S., Debets R., Gratama J.W. (2011). Immune responses to transgene and retroviral vector in patients treated with ex vivo-engineered T cells. Blood.

[21] Beatty G.L., Haas A.R., Maus M.V., Torigian D.A., Soulen M.C., Plesa G., Chew A., Zhao Y., Levine B.L., Albelda S.M. (2014). Mesothelin-specific chimeric antigen receptor mRNA-engineered T cells induce anti-tumor activity in solid malignancies. Cancer Immunol. Res..

[22] You F., Jiang L., Zhang B., Lu Q., Zhou Q., Liao X., Wu H., Du K., Zhu Y., Meng H. (2016). Phase 1 clinical trial demonstrated that MUC1 positive metastatic seminal vesicle cancer can be effectively eradicated by modified Anti-MUC1 chimeric antigen receptor transduced T cells. Sci. China Life Sci..

[23] Zhang C., Wang Z., Yang Z., Wang M., Li S., Li Y., Zhang R., Xiong Z., Wei Z., Shen J. (2017). Phase I Escalating-Dose Trial of CAR-T Therapy Targeting CEA+ Metastatic Colorectal Cancers. Mol. Ther..

[24] Katz S.C., Burga R.A., McCormack E., Wang L.J., Mooring W., Point G.R., Khare P.D., Thorn M., Ma Q., Stainken B.F. (2015). Phase I Hepatic Immunotherapy for Metastases Study of Intra-Arterial Chimeric Antigen Receptor-Modified T-cell Therapy for CEA+ Liver Metastases. Clin. Cancer Res..

[25] Junghans R.P., Ma Q., Rathore R., Gomes E.M., Bais A.J., Lo A.S., Abedi M., Davies R.A., Cabral H.J., Al-Homsi A.S. (2016). Phase I Trial of Anti-PSMA Designer CAR-T Cells in Prostate Cancer: Possible Role for Interacting Interleukin 2-T Cell Pharmacodynamics as a Determinant of Clinical Response. Prostate.

[26] Tanyi J.L., Stashwick C., Plesa G., Morgan M.A., Porter D., Maus M.V., June C.H. (2017). Possible Compartmental Cytokine Release Syndrome in a Patient with Recurrent Ovarian Cancer After Treatment With Mesothelin-targeted CAR-T Cells. J. Immunother..

[27] Brown C.E., Alizadeh D., Starr R., Weng L., Wagner J.R., Naranjo A., Ostberg J.R., Blanchard M.S., Kilpatrick J., Simpson J. (2016). Regression of Glioblastoma after Chimeric Antigen Receptor T-Cell Therapy. N. Engl. J. Med..

[28] Tchou J., Zhao Y., Levine B.L., Zhang P.J., Davis M.M., Melenhorst J.J., Kulikovskaya I., Brennan A.L., Liu X., Lacey S.F. (2017). Safety and Efficacy of Intratumoral Injections of Chimeric Antigen Receptor (CAR) T Cells in Metastatic Breast Cancer. Cancer Immunol. Res..

[29] Hege K.M., Bergsland E.K., Fisher G.A., Nemunaitis J.J., Warren R.S., McArthur J.G., Lin A.A., Schlom J., June C.H., Sherwin S.A. (2017). Safety, tumor trafficking and immunogenicity of chimeric antigen receptor (CAR)-T cells specific for TAG-72 in colorectal cancer. J. Immunother. Cancer.

[30] Ali S.A., Shi V., Maric I., Wang M., Stroncek D.F., Rose J.J., Brudno J.N., Stetler-Stevenson M., Feldman S.A., Hansen B.G. (2016). T cells expressing an anti-B-cell maturation antigen chimeric antigen receptor cause remissions of multiple myeloma. Blood.

[31] Maus M.V., Haas A.R., Beatty G.L., Albelda S.M., Levine B.L., Liu X., Zhao Y., Kalos M., June C.H. (2013). T cells expressing chimeric antigen receptors can cause anaphylaxis in humans. Cancer Immunol. Res..

[32] Thistlethwaite F.C., Gilham D.E., Guest R.D., Rothwell D.G., Pillai M., Burt D.J., Byatte A.J., Kirillova N., Valle J.W., Sharma S.K. (2017). The clinical efficacy of first-generation carcinoembryonic antigen (CEACAM5)-specific CAR T cells is limited by poor persistence and transient pre-conditioning-dependent respiratory toxicity. Cancer Immunol. Immunother..

[33] Lamers C.H., Sleijfer S., van Steenbergen S., van Elzakker P., van Krimpen B., Groot C., Vulto A., den Bakker M., Oosterwijk E., Debets R. (2013). Treatment of metastatic renal cell carcinoma with CAIX CAR-engineered T cells: Clinical evaluation and management of on-target toxicity. Mol. Ther..

[34] Weng J., Lai P., Qin L., Lai Y., Jiang Z., Luo C., Huang X., Wu S., Shao D., Deng C. (2018). A novel generation 1928zT2 CAR T cells induce remission in extramedullary relapse of acute lymphoblastic leukemia. J. Hematol. Oncol..

[35] Gardner R., Wu D., Cherian S., Fang M., Hanafi L.A., Finney O., Smithers H., Jensen M.C., Riddell S.R., Maloney D.G. (2016). Acquisition of a CD19-negative myeloid phenotype allows immune escape of MLL-rearranged B-ALL from CD19 CAR-T cell therapy. Blood.

[36] Zhu Y.M., Wu Z., Tan Y.P., Du Y.Y., Liu Z., Ou R.M., Liu S., Pu C.F., Jiang J., Wang J.P. (2016). Anti-CD19 chimeric antigen receptor T-cell therapy for adult Philadelphia chromosome-positive acute lymphoblastic leukemia: Two case reports. Medicine.

[37] Wang C.M., Wu Z.Q., Wang Y., Guo Y.L., Dai H.R., Wang X.H., Li X., Zhang Y.J., Zhang W.Y., Chen M.X. (2017). Autologous T Cells Expressing CD30 Chimeric Antigen Receptors for Relapsed or Refractory Hodgkin Lymphoma: An Open-Label Phase I Trial. Clin. Cancer Res..

[38] Callahan C., Baniewicz D., Ely B. (2017). CAR T-Cell Therapy: Pediatric Patients With Relapsed and Refractory Acute Lymphoblastic Leukemia. Clin. J. Oncol. Nurs..

[39] Turtle C.J., Hanafi L.A., Berger C., Gooley T.A., Cherian S., Hudecek M., Sommermeyer D., Melville K., Pender B., Budiarto T.M. (2016). CD19 CAR-T cells of defined CD4+:CD8+ composition in adult B cell ALL patients. J. Clin. Investig..

[40] Wei G., Hu Y., Pu C., Yu J., Luo Y., Shi J., Cui Q., Wu W., Wang J., Xiao L. (2018). CD19 targeted CAR-T therapy versus chemotherapy in re-induction treatment of refractory/relapsed acute lymphoblastic leukemia: Results of a case-controlled study. Ann. Hematol..

[41] Wang Y.Y., Shi X.D., Li J.H., Zhang X.X., Li J.J., Feng S.Q. (2017). Clinical Observation of CD19 CAR T Cell Therapy in Children Relapse B Lymphocytic Leukemia/Lymphoma. J. Inn. Mong. Med. Univ..

[42] Fry T.J., Shah N.N., Orentas R.J., Stetler-Stevenson M., Yuan C.M., Ramakrishna S., Wolters P., Martin S., Delbrook C., Yates B. (2018). CD22-targeted CAR T cells induce remission in B-ALL that is naive or resistant to CD19-targeted CAR immunotherapy. Nat. Med..

[43] Kochenderfer J.N., Dudley M.E., Kassim S.H., Somerville R.P., Carpenter R.O., Stetler-Stevenson M., Yang J.C., Phan G.Q., Hughes M.S., Sherry R.M. (2015). Chemotherapy-refractory diffuse large B-cell lymphoma and indolent B-cell malignancies can be effectively treated with autologous T cells expressing an anti-CD19 chimeric antigen receptor. J. Clin. Oncol..

[44] Maude S.L., Frey N., Shaw P.A., Aplenc R., Barrett D.M., Bunin N.J., Chew A., Gonzalez V.E., Zheng Z., Lacey S.F. (2014). Chimeric antigen receptor T cells for sustained remissions in leukemia. N. Engl. J. Med..

[45] Porter D.L., Hwang W.T., Frey N.V., Lacey S.F., Shaw P.A., Loren A.W., Bagg A., Marcucci K.T., Shen A., Gonzalez V. (2015). Chimeric antigen receptor T cells persist and induce sustained remissions in relapsed refractory chronic lymphocytic leukemia. Sci. Transl. Med..

[46] Ramos C.A., Ballard B., Zhang H., Dakhova O., Gee A.P., Mei Z., Bilgi M., Wu M.F., Liu H., Grilley B. (2017). Clinical and immunological responses after CD30-specific chimeric antigen receptor-redirected lymphocytes. J. Clin. Investig..

[47] Chen Y., Cheng Y., Suo P., Yan C., Wang Y., Chen Y., Han W., Xu L., Zhang X., Liu K. (2017). Donor-derived CD19-targeted T cell infusion induces minimal residual disease-negative remission in relapsed B-cell acute lymphoblastic leukaemia with no response to donor lymphocyte infusions after haploidentical haematopoietic stem cell transplantation. Br. J. Haematol..

[48] Kochenderfer J.N., Dudley M.E., Carpenter R.O., Kassim S.H., Rose J.J., Telford W.G., Hakim F.T., Halverson D.C., Fowler D.H., Hardy N.M. (2013). Donor-derived CD19-targeted T cells cause regression of malignancy persisting after allogeneic hematopoietic stem cell transplantation. Blood.

[49] Wang Y., Zhang W.Y., Han Q.W., Liu Y., Dai H.R., Guo Y.L., Bo J., Fan H., Zhang Y., Zhang Y.J. (2014). Effective response and delayed toxicities of refractory advanced diffuse large B-cell lymphoma treated by CD20-directed chimeric antigen receptor-modified T cells. Clin. Immunol..

[50] Davila M.L., Riviere I., Wang X., Bartido S., Park J., Curran K., Chung S.S., Stefanski J., Borquez-Ojeda O., Olszewska M. (2014). Efficacy and toxicity management of 19-28z CAR T cell therapy in B cell acute lymphoblastic leukemia. Sci. Transl. Med..

[51] Pan J., Yang J.F., Deng B.P., Zhao X.J., Zhang X., Lin Y.H., Wu Y.N., Deng Z.L., Zhang Y.L., Liu S.H. (2017). High efficacy and safety of low-dose CD19-directed CAR-T cell therapy in 51 refractory or relapsed B acute lymphoblastic leukemia patients. Leukemia.

[52] Fraietta J.A., Beckwith K.A., Patel P.R., Ruella M., Zheng Z., Barrett D.M., Lacey S.F., Melenhorst J.J., McGettigan S.E., Cook D.R. (2016). Ibrutinib enhances chimeric antigen receptor T-cell engraftment and efficacy in leukemia. Blood.

[53] Turtle C.J., Hanafi L.A., Berger C., Hudecek M., Pender B., Robinson E., Hawkins R., Chaney C., Cherian S., Chen X. (2016). Immunotherapy of non-Hodgkin’s lymphoma with a defined ratio of CD8+ and CD4+ CD19-specific chimeric antigen receptor-modified T cells. Sci. Transl. Med..

[54] Cruz C.R., Micklethwaite K.P., Savoldo B., Ramos C.A., Lam S., Ku S., Diouf O., Liu E., Barrett A.J., Ito S. (2013). Infusion of donor-derived CD19-redirected virus-specific T cells for B-cell malignancies relapsed after allogeneic stem cell transplant: A phase 1 study. Blood.

[55] Gardner R.A., Finney O., Annesley C., Brakke H., Summers C., Leger K., Bleakley M., Brown C., Mgebroff S., Kelly-Spratt K.S. (2017). Intent-to-treat leukemia remission by CD19 CAR T cells of defined formulation and dose in children and young adults. Blood.

[56] Hay K.A., Hanafi L.A., Li D., Gust J., Liles W.C., Wurfel M.M., López J.A., Chen J., Chung D., Harju-Baker S. (2017). Kinetics and biomarkers of severe cytokine release syndrome after CD19 chimeric antigen receptor-modified T-cell therapy. Blood.

[57] Park J.H., Rivière I., Gonen M., Wang X., Sénéchal B., Curran K.J., Sauter C., Wang Y., Santomasso B., Mead E. (2018). Long-Term Follow-up of CD19 CAR Therapy in Acute Lymphoblastic Leukemia. N. Engl. J. Med..

[58] Qasim W., Zhan H., Samarasinghe S., Adams S., Amrolia P., Stafford S., Butler K., Rivat C., Wright G., Somana K. (2017). Molecular remission of infant B-ALL after infusion of universal TALEN gene-edited CAR T cells. Sci. Transl. Med..

[59] Ritchie D.S., Neeson P.J., Khot A., Peinert S., Tai T., Tainton K., Chen K., Shin M., Wall D.M., Hönemann D. (2013). Persistence and efficacy of second generation CAR T cell against the LeY antigen in acute myeloid leukemia. Mol. Ther..

[60] Locke F.L., Neelapu S.S., Bartlett N.L., Siddiqi T., Chavez J.C., Hosing C.M., Ghobadi A., Budde L.E., Bot A., Rossi J.M. (2017). Phase 1 Results of ZUMA-1: A Multicenter Study of KTE-C19 Anti-CD19 CAR T Cell Therapy in Refractory Aggressive Lymphoma. Mol. Ther..

[61] Lee D.W., Kochenderfer J.N., Stetler-Stevenson M., Cui Y.K., Delbrook C., Feldman S.A., Fry T.J., Orentas R., Sabatino M., Shah N.N. (2015). T cells expressing CD19 chimeric antigen receptors for acute lymphoblastic leukaemia in children and young adults: A phase 1 dose-escalation trial. Lancet.

[62] Kalos M., Levine B.L., Porter D.L., Katz S., Grupp S.A., Bagg A., June C.H. (2011). T cells with chimeric antigen receptors have potent antitumor effects and can establish memory in patients with advanced leukemia. Sci. Transl. Med..

[63] Maude S.L., Laetsch T.W., Buechner J., Rives S., Boyer M., Bittencourt H., Bader P., Verneris M.R., Stefanski H.E., Myers G.D. (2018). Tisagenlecleucel in Children and Young Adults with B-Cell Lymphoblastic Leukemia. N. Engl. J. Med..

[64] Dai H., Zhang W., Li X., Han Q., Guo Y., Zhang Y., Wang Y., Wang C., Shi F., Zhang Y. (2015). Tolerance and efficacy of autologous or donor-derived T cells expressing CD19 chimeric antigen receptors in adult B-ALL with extramedullary leukemia. Oncoimmunology.

[65] Neelapu S.S., Locke F.L., Bartlett N.L., Lekakis L.J., Miklos D.B., Jacobson C.A., Braunschweig I., Oluwole O.O., Siddiqi T., Lin Y. (2017). Axicabtagene Ciloleucel CAR T-Cell Therapy in Refractory Large B-Cell Lymphoma. N. Engl. J. Med..

[66] Priceman S.J., Forman S.J., Brown C.E. (2015). Smart CARs engineered for cancer immunotherapy. Curr. Opin. Oncol..

[67] Kakarla S., Gottschalk S. (2014). CAR T cells for solid tumors: Armed and ready to go?. Cancer J..

[68] Yu W.L., Hua Z.C. (2018). Evaluation of effectiveness of granulocyte-macrophage colony-stimulating factor therapy to cancer patients after chemotherapy: A meta-analysis. Oncotarget.

[69] Whiting P.F., Rutjes A.W., Westwood M.E., Mallett S., Deeks J.J., Reitsma J.B., Leeflang M.M., Sterne J.A., Bossuyt P.M., QUADAS-2 Group (2011). QUADAS-2: A revised tool for the quality assessment of diagnostic accuracy studies. Ann. Intern. Med..

[70] QUADAS-2 Manual. http://www.bris.ac.uk/quadas/quadas-2/.

